# Leigh Syndrome Caused by Compound Heterozygous Variants c.1162A_C and c.1138G_C in the NDUFV1 Gene: A Case Report

**DOI:** 10.7759/cureus.71127

**Published:** 2024-10-09

**Authors:** Josef Finsterer

**Affiliations:** 1 Neurology, Neurology and Neurophysiology Center, Vienna, AUT

**Keywords:** complex-i deficiency, leigh-like syndrome, mitochondrial dna, ndufv1, respiratory chain

## Abstract

Early-onset Leigh syndrome is usually a genetically and phenotypically heterogeneous, severe, rapidly progressive mitochondrial disorder with a fatal outcome. Leigh syndrome is genetically heterogeneous as it is based on mutations in mtDNA or nDNA genes, which mostly encode subunits of respiratory chain complexes or assembly factors. It is phenotypically heterogeneous because it is genetically heterogeneous and due to the peculiarities of mitochondrial genetics. One of the more than 100 mutated genes responsible for Leigh syndrome is NDUFV1. Here we present the case of an infant with Leigh syndrome who suffered from a novel heterozygous variant of the NDUFV1 gene, which is phenotypically characterized by a number of previously unknown features.

The patient was a four-month-old girl with Leigh syndrome due to the compound heterozygous variants c.1162+4A>C (previously described, inherited from the mother) and c.1138G>C (novel, inherited from the father) in NDUFV1. The mutation c.1162+4A>C is a non-canonical splice site variant that has been demonstrated to result in loss of function. The bioinformatic analysis supports that the missense variant c. 1138G>C has a deleterious effect on protein structure or function. The mutations manifested phenotypically with typical cerebral lesions on imaging, developmental delay, cognitive decline, epileptiform discharges in the electroencephalography without seizures, atrioventricular (AV) block II, agenesis of a subclavian vein, right heart failure, patent foramen ovale, pulmonary hypertension, hypoaldosteronism, and abdominal hernias. Within five weeks of hospitalization, the disease took a progressive course, and the patient died of infectious complications despite maximum treatment.

This case shows that the described new heterozygous variant in NDUFV1 can occur with previously undescribed phenotypic features. It is important to diagnose mitochondrial disorders due to NDUFV1 mutations early in order not to miss the time for appropriate symptomatic treatment.

## Introduction

Leigh syndrome, also known as subacute necrotizing encephalomyelopathy, is a devastating primary multisystem mitochondrial disorder (MID) that typically manifests in infancy or early childhood, but cases with late-onset have also been reported [1]. Since its first description by Denis Archibald Leigh in 1951, the disease has evolved from a postmortem diagnosis defined strictly by histopathologic observations to a clinical entity with indicative laboratory, radiologic, and genetic findings [1]. The disease is radiologically characterized by symmetrical lesions typically extending from the basal ganglia and thalamus through brainstem structures to the cerebellum and posterior columns on magnetic resonance imaging (MRI), a clinical course with rapid deterioration of cognitive and motor functions, and mutations in genes affecting mitochondrial metabolism, signal transduction, or morphology [1]. Central nervous system (CNS) lesions are histologically characterized by capillary proliferation, gliosis, severe neuronal loss, and relative preservation of astrocytes [2]. The most commonly affected organ is the CNS, but in general, all other organs and tissues can be affected, especially skeletal muscle, heart, gastrointestinal tract, and endocrine organs [3]. In the CNS, Leigh syndrome can manifest as psychomotor retardation, seizures, nystagmus, ophthalmoparesis, optic atrophy, ataxia, dystonia, or central respiratory failure [3].

Genetically, Leigh syndrome is due to mutations in more than 100 nuclear genes and at least 16 mtDNA genes and exhibits great clinical, immunohistochemical, and biochemical heterogeneity [2]. According to pathophysiological criteria, the phenotypes of Leigh syndrome are divided into the following categories: disorders of subunits and assembly factors, disorders of pyruvate metabolism and vitamin and cofactor transport metabolism, disorders of mtDNA maintenance, defects in mitochondrial gene expression, protein quality control and lipid remodeling, dynamics, and toxicity [2]. Most phenotypes of Leigh syndrome respond only to symptomatic therapy, but some of them are treatable [2]. Leigh syndrome due to the heterozygous variants c.1162+4A>C and c.1138G>C in NDUFV1 has not yet been reported. The NDUFV1 gene encodes a subunit of the enzyme NADH-ubiquinone oxidoreductase (complex I), a large, multimeric protein. It is the first enzyme complex in the mitochondrial electron transport chain and catalyzes the transfer of electrons from NADH to the electron acceptor ubiquinone. The proton gradient created by the electron transfer drives the conversion of ADP to ATP. This gene is a central subunit and is conserved in prokaryotes and eukaryotes. The human ortholog of this protein has been characterized. It possesses consensus motifs for NADH, flavin mononucleotide, and iron-sulfur binding sites and is involved in the oxidation of NADH as part of the dehydrogenase module of complex I. In humans, deficiencies in complex I are associated with myopathies, encephalomyopathies, and neurodegenerative disorders.

## Case presentation

The patient was a four-month-old girl with Leigh syndrome that phenotypically manifested with encephalopathy, epilepsy, congenital nystagmus, aberrant subclavian artery, inguinal and umbilical hernia, pulmonary artery hypertension, patent foramen ovale, right ventricular hypertrophy, normocytic anemia, syndrome of inappropriate antidiuretic hormone secretion (SIADH), and lactic acidosis.

The patient was a baby conceived by intrauterine insemination, delivered by a 36-year-old gravida 2 primi 2 at term by repeat cesarean section. The pregnancy was complicated by maternal nausea (requiring ondansetron), a mild urinary tract infection (requiring antibiotics), and symptoms of upper respiratory tract infection (with paracetamol pro re nata (PRN, as needed)). The pregnancy was otherwise uncomplicated with normal prenatal imaging and normal non-invasive prenatal testing (NIPT). The newborn did not require a stay in the neonatal intensive care unit (NICU). Postnatally, however, she was found to have an umbilical and inguinal hernia. She also had an asymptomatic heart murmur. Because of this, and in preparation for surgical repair of the hernia, she consulted the cardiology service, who found an aberrant subclavian vein and patent foramen ovale on echocardiography. During an upper respiratory infection (URI) with *Pneumocystis carinii* pneumonia at two months of age, the patient was also found to have horizontal nystagmus. The ophthalmologic examination confirmed the horizontal nystagmus but considered it normal for her age, and no further examination, including an MRI of the brain, was necessary. Since then, she had been in her normal state of health.

Family history was positive for myopia (mother), asymptomatic c.3922G>T BRCA2 mutation (father, sister), Cornelia De Lange syndrome (microcephaly, dysmorphism, intellectual decline, short stature, hypertrichosis, synophris, abnormal hands and feet, cardiac and renal malformations) due to the probable pathogenic variant c.64 C>G in RAD21 (sister), breast cancer in the maternal grandmother, cardiac arrhythmia and COVID-19 (maternal grandfather), and Hodgkin's lymphoma (paternal uncle). As the risk of recurrence of the RAD21 mutation was considered low, the index patient had not undergone an amniocentesis. The parents were not consanguineous.

At four months and one week of age, she was admitted due to generalized weakness (which manifested in an altered cry), lethargy, altered mental status (AMS), lack of responsiveness, and cyanosis (oxygen (O_2_) saturation 85%) following a URI symptom. Four days prior to admission, she started sleeping through the night, but the parents were not very concerned about this 3as babies of this age usually sleep more. The patient was intubated and sedated to protect her airway. Cerebral computed tomography revealed no evidence of hemorrhage, mass, or midline shift. Laboratory values showed hyponatremia of 125 mmol/l, which was treated with a bolus of hypertonic saline. Hydrocortisone was administered on suspicion of a metabolic disorder. After a complete septic workup, including lumbar puncture (LP), ceftriaxone was administered. The patient was also given a bolus of levetiracetam (28 mg/kg) as a seizure was suspected as a possible cause.

**Figure 1:**
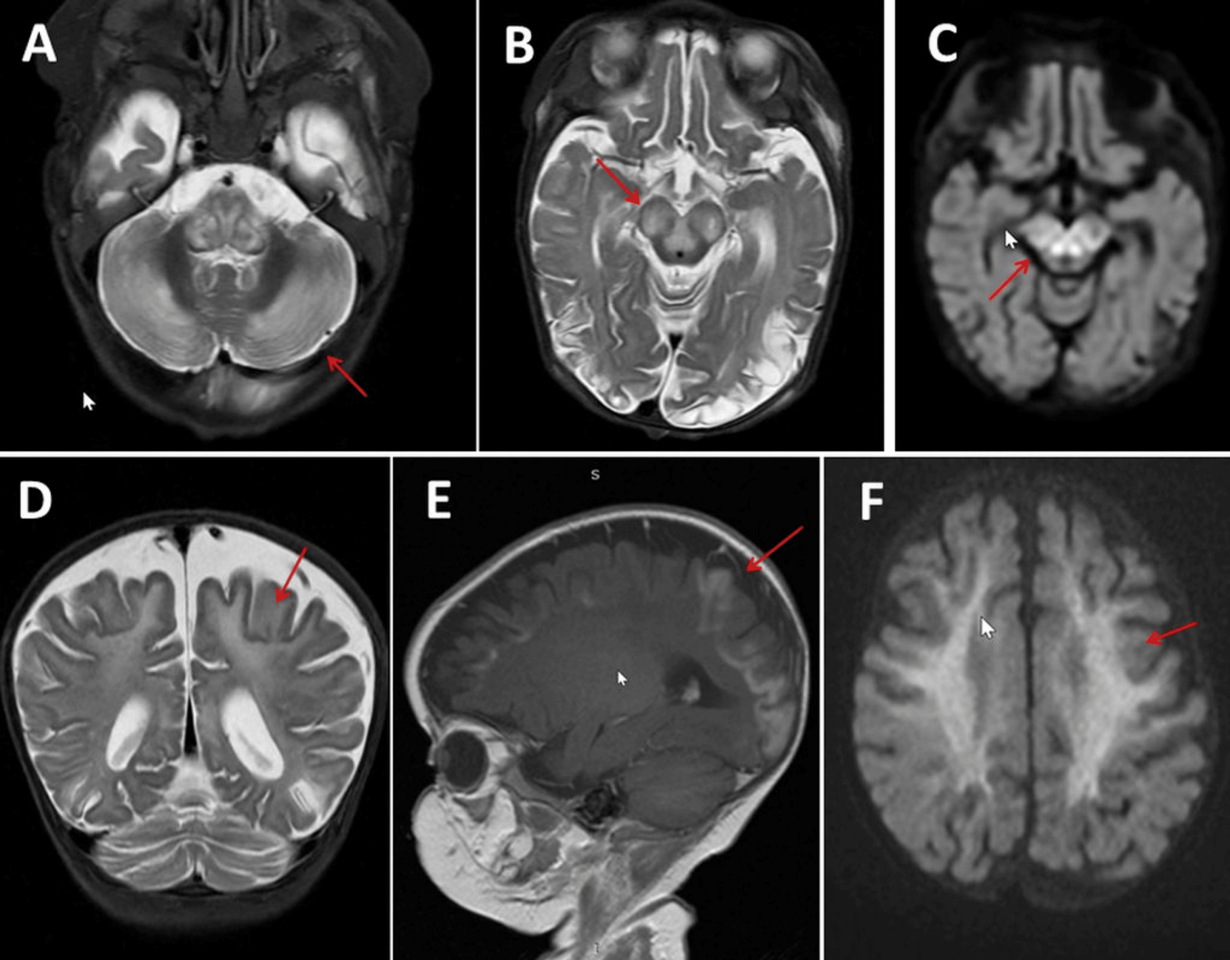
Cerebral magnetic resonance imaging showing symmetrical T2-hyperintense lesions in the cerebellar hemispheres (panel A), symmetric T2-hyperintense lesions in the crura cerebri (panel B), symmetric diffusion-weighted imaging (DWI) -hyperintense lesions in the midbrain (panel C), symmetrical deep and subcortical white matter lesions (panel D), T1-hyperintense lesions in the occipital lobe (panel E), and symmetrical diffuse DWI-hyperintensities in the white matter (panel F)

She was then transferred to the pediatric intensive care unit (PICU). The course in the PICU was complicated by hypotension despite intravenous fluid bolus administration. Norepinephrine (0.05 mcg/kg/min) was administered, and the patient received hydrocortisone to treat the refractory hypotension. As the ECG findings indicated type 2 atrioventricular (AV) block II (Mobitz II), echocardiography was performed immediately, which revealed severely impaired right ventricular systolic function, mild right ventricular hypertrophy, and pulmonary hypertension (Figure 1). At the same time, troponin and pro-brain natriuretic peptide (pro-BNP) had risen significantly to 9.8 ng/l and 1554 ng/l, respectively. The patient also had metabolic acidosis with a serum lactate of 12.05 mmol/l. The patient was also receiving milrinone and nitric oxide. As the etiology of the hypotensive episode and the acute onset of right heart strain were still unclear, a second echocardiogram was performed, which showed no evidence of myocarditis. Computed tomography and angiography of the chest revealed no evidence of pulmonary embolism. Despite the unclear etiology of right ventricular systolic dysfunction and arterial hypotension, the patient improved clinically, troponins and pro-BNP decreased, and further echocardiography showed near-normal right ventricular systolic function compared to previous examinations.

**Figure 2:**
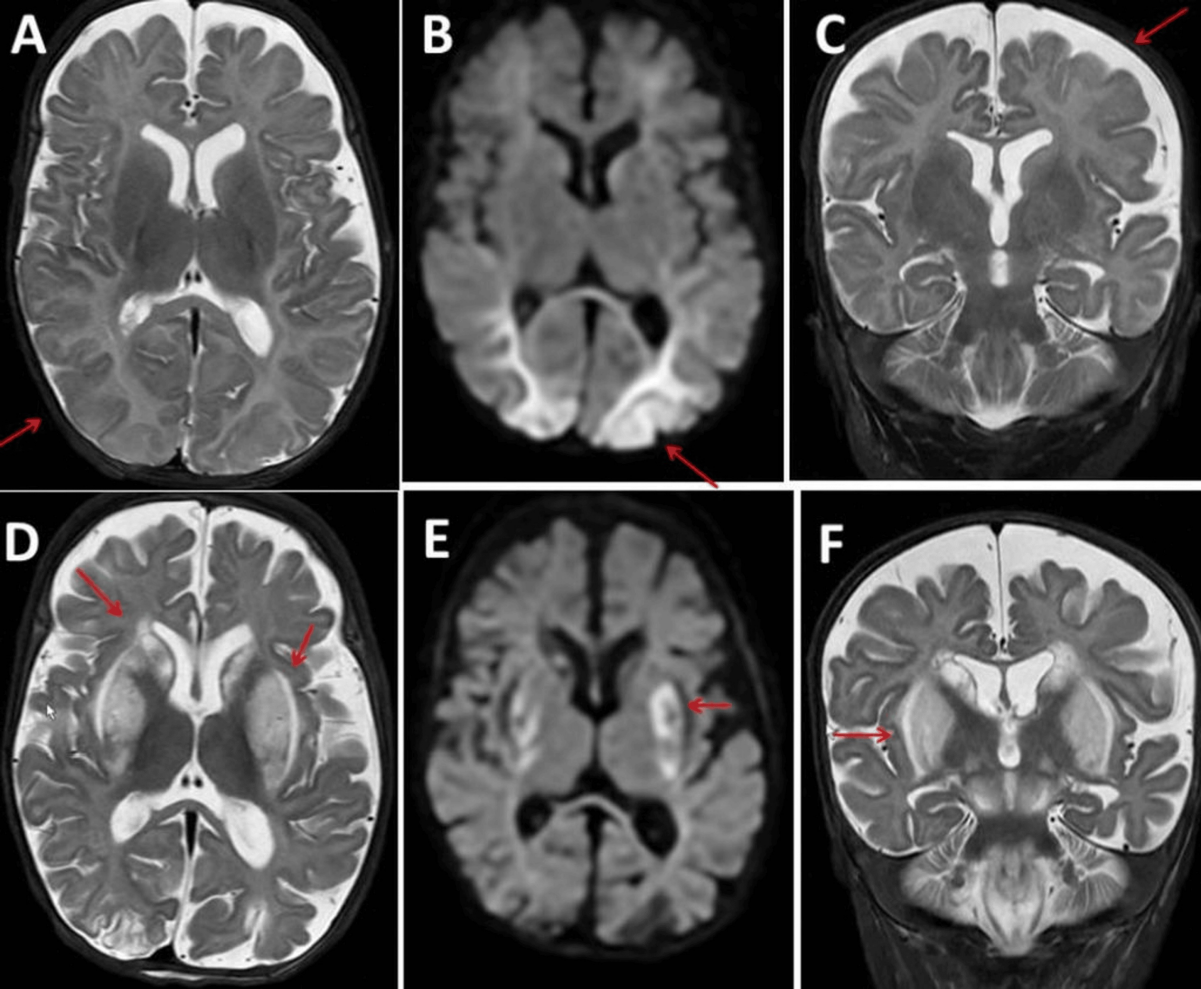
Cerebral magnetic resonance imaging shortly after admission (panels A to C) compared with images from four weeks later (panels D to F) showing progression of axial and coronary T2 hyperintensities in the basal ganglia (panels A and D and panels C and F) and new diffusion-weighted imaging (DWI) hyperintensities in the basal ganglia (panel E) but regression of DWI hyperintensities in the occipital lobes (panel B)

Cerebral MRI shortly after admission showed diffuse areas of restricted diffusion, most prominent in the parietal-occipital region but also in the bifrontal regions and posterior temporal regions (Figure 1). Expansion was noted at the boundary between gray and white matter (Figure 2). There were signaling abnormalities extending to the cerebellar hemispheres and vermis, as well as along the medulla and cervical spinal cord (Figure 2). These abnormalities progressed within one month of hospitalization (Figure 2). Differential diagnoses considered were inflammatory disease (e.g., encephalitis), hypoxic injury, metabolic disease (e.g., osmotic myelinolysis), or mitochondrial dysfunction. Genetic testing for MID revealed the heterozygous variants c.1162+4A>C (inherited from the mother) and c.1138G>C (inherited from the father) in NDUFV1. Electroencephalography (EEG) showed left temporal spikes with a negative history of seizures. The cerebrospinal fluid (CSF) findings were inconclusive. The patient was weaned off norepinephrine and could be extubated.

Four days after extubation, the patient was put back on oxygen due to intermittent irregular breathing. Due to an episode of cyanosis and apnea, she required emergency intubation one day later and was placed on a neurally adjusted ventilator assist (NAVA), but had to be switched to pressure-regulated volume control (PRVC) due to episodes of apnea and flaccid paralysis. White blood cell (WBC) count rose to 20 106/l and procalcitonin was slightly elevated and there was concern for bowel obstruction. She was treated with intravenous immunoglobulins and glucocorticoids as the culture remained negative. One day later, an ultrasound revealed an occluded thrombus in the left common iliac vein, external iliac vein, common femoral vein, and proximal femoral vein, so enoxaparin (6mg every 12h) was administered.

**Figure 3:**
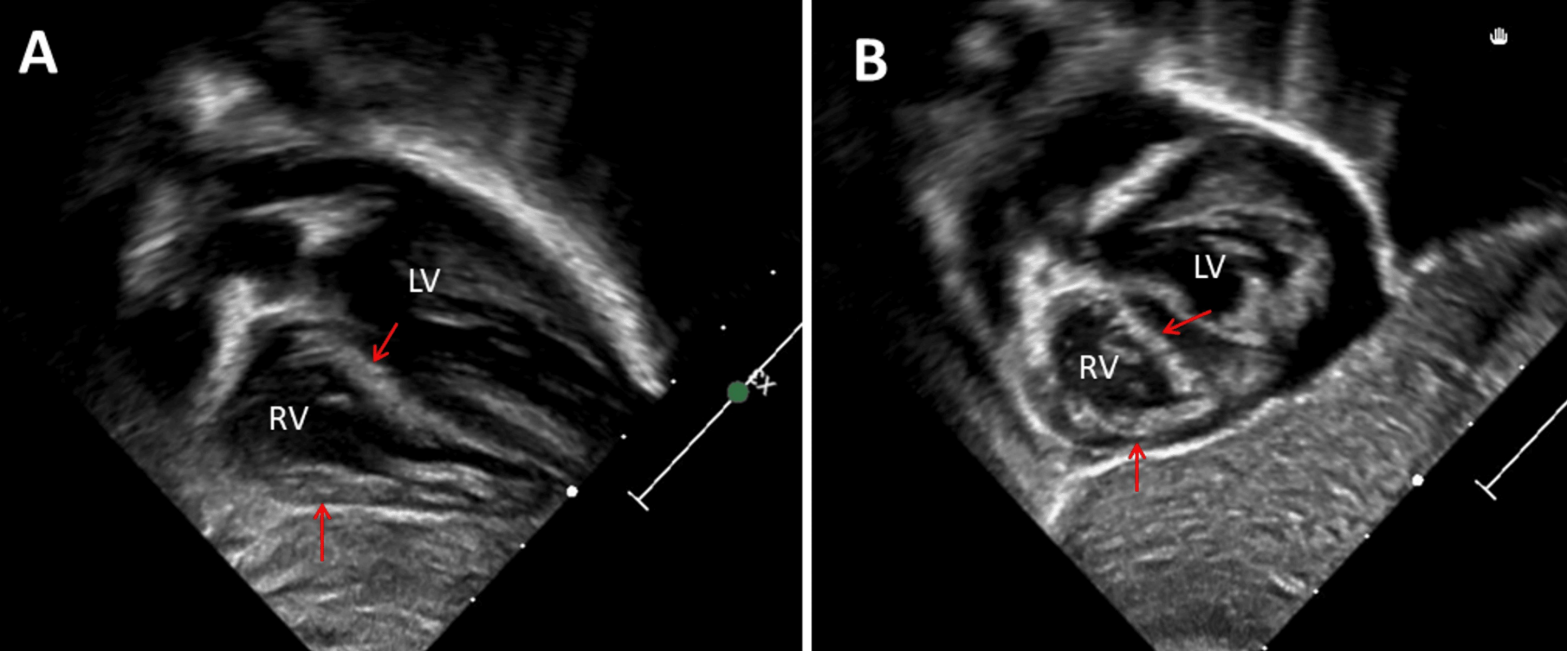
Transthoracic echocardiography showing right ventricular hypertrophy, which has not been found on previous examinations, and mild enlargement of the right ventricle (panels A and B).

Four days later, feeding was resumed via the nasogastric tube. Despite the discontinuation of pressure medication and sedatives, she only showed abdominal and lower limb movements. The carbon monoxide level was persistently high. The patient was evaluated with regard to palliative care or tracheostomy and G-tube. One day later, higher ventilation settings were required. A repeat MRI scan of the brain and spine showed progression of the previously described lesions (Figure 2). Echocardiography showed mild right ventricular hypertrophy, which had not been present previously (Figure 3). Five weeks after admission, the patient died. The last medication until death included acetylcysteine, albuterol, amlodipine, chlorhexidine, cholecalciferol, enoxaparin, famotidine, ipratropium, L-carnitine, ubiquinol, vitamin B-50 complex, dexmedetomidine, acetaminophen, lidocaine, lorazepam, morphine, naloxone and nystatin. An autopsy was not performed.

## Discussion

The presented patient is of interest for Leigh syndrome due to a novel combination of the two heterozygous variants c.1162+4A>C and c.1138G>C in NDUFV1, of which the variant c.1138G>C is novel. Phenotypically, the index patient mainly showed a CNS disorder with developmental delay, cognitive impairment, epileptiform discharges on EEG without seizures, symmetrical gray and white matter lesions, nystagmus, and spinal cord involvement. Cardiac involvement manifested as AV block II, agenesis of the subclavian vein, right heart failure, patent foramen ovale, pulmonary hypertension, and right ventricular hypertrophy. Endocrine involvement manifested as hypoaldosteronism and SIADH. Gastrointestinal involvement manifested as umbilical and inguinal hernias. The patient was also one of the few NDUFV1 nutation carriers in the literature with lactic acidosis. Whether anemia and hepatopathy are due to the ongoing infection or treatment during the PICU stay remains speculative, but it cannot be ruled out that anemia and hepatopathy are also phenotypic features of Leigh syndrome as previously reported [4,5].

Leigh syndrome due to mutations in NDUFV1 had been diagnosed in at least 44 patients by June 2024 [6]. Depending on the underlying genotype, the phenotypic expression of these NDUFV1 variants can be quite heterogeneous. The most frequently affected organs are the brain, skeletal muscles, and the gastrointestinal tract (Table 1). The most common CNS abnormalities include spasticity, psychomotor regression, hypotonia or floppiness, and nystagmus. The most common skeletal muscle abnormalities include myopathy of the limbs, strabismus, or ptosis. Common gastrointestinal manifestations of the disease include vomiting, poor feeding or failure to thrive, and dysphagia. A number of patients also experience movement disorders such as dystonia or tremor. Lactic acidosis has been reported in only a few patients (Table 1). The course of the disease can range from a rapidly progressive CNS disease with early death to a slowly progressive disease with death in adulthood [6]. Patients die from intractable seizures, hypoventilation, and central apnea, infectious diseases due to aspiration or sepsis [7-20].

**Table 1:** Phenotypic features among 44 patients with Leigh syndrome due to pathogenic variants in NDUFV1 Adapted from Mahesan et al.'s study titled *NDUFV1-related mitochondrial complex-1 disorders: a retrospective case series and literature review *[6]

Organ	Abnormality	Frequency (n)
Central nervous system	Myoclonic epilepsy	3
	Seizures	8
	Psychomotor retardation	8
	Hypotonia, floppiness	13
	Spasticity	24
	Ataxia	7
	Dystonia	5
	Hypoventilation, apnea	5
	Psychomotor regression	17
	Involuntary movements	1
	Irritability	10
	Nystagmus	11
	Tremor, head titubation	4
	Learning difficulty	2
	Dysarthria	5
	Cognitive impairment	2
Ophthalmologic	Optic atrophy	6
	Myopia	1
Ototologic	Deafness	1
Gastrointestinal	Vomiting	8
	Dysphagia	6
	Failure to thrive, poor feeding	7
	Hepatomegaly	2
Striated muscle	Ptosis	4
	External ophthalmoplegia	1
	Strabismus	12
	Myopathy	13
Peripheral nerves	Neuropathy	1
Skeleton	Macrocephaly	1
	Scoliosis	1
Other	Lethargy	2
	Lactic acidosis	1
	Excessive crying	1

Most of the phenotypic features described in the index patient have been described previously. However, some features are new, such as hypoaldosteronism, hernias, patent foramen ovale, cardiac arrhythmias (e.g., AV-block II), and pulmonary hypertension. The discrepancy may be due to the fact that not all previously reported patients may have been systematically and prospectively screened for multisystem involvement, including cardiac involvement. Poor phenotypic characterization may also be due to poor patient or parental compliance and inadequate follow-up. Only if these patients are followed up closely can the course of the individual disease be adequately assessed. As for the genetic cause of the index patient's phenotype, the c.1138G>C variant (inherited from the father) has not been reported previously, but the c.1162+4A>C variant (inherited from the mother) has been reported once [15]. This patient, like the index patient, was also compound heterozygous, but the second mutation was the c.640G>A variant [15]. Phenotypically, this patient presented with Leigh syndrome with febrile seizures, dystonia, left hemiparesis, tremor, dysarthria, ptosis, and dysphagia [15]. This particular patient benefited from thiamine, coenzyme Q, L-carnitine, and biotin [15].

## Conclusions

In summary, this case shows that the described novel combination of compound heterozygous variants in NDUFV1 can manifest with new phenotypic features not previously described in NDUFV1 mutation carriers, such as hypoaldosteronism, hernias, patent foramen ovale, cardiac arrhythmias (e.g., AV-block II), right ventricular hypertrophy, and pulmonary hypertension. It is important to diagnose MIDs due to NDUFV1 mutations early so as not to miss time for appropriate treatment. Carriers of an NDUFV1 variant require regular and close follow-up as most cases, especially those with poor outcomes, have a rapidly progressive course that requires full attention, vigilance, and monitoring of treating physicians and caring parents. Future research could focus on the consequences of the described NDUFV1 variant in relation to the function of complex I of the respiratory chain and the impairment of ATP production due to this mutation. Research activities should also focus on cardiac involvement in Leigh syndrome, particularly of the right heart, and pulmonary hypertension. 
